# A hydrolase from *Serratia liquefaciens* IMD717 with esterase, dehalogenase and *N*-deformylase activities

**DOI:** 10.1007/s00253-026-13829-7

**Published:** 2026-04-20

**Authors:** Mohd Faheem Khan, Periklis Karamanis, Beate Koksch, Cormac D. Murphy

**Affiliations:** 1 School of Biomolecular and Biomedical Science, University College Dublin, Belfield, Dublin 4 Ireland; 2 School of Chemistry, University College Dublin, Belfield, Dublin 4 Ireland; 3 Institute of Chemistry and Biochemistry, Freie Universität Berlin, Arnimallee 20, Berlin, 14195 Germany; 4 UCD Conway Institute, University College Dublin, Belfield, Dublin 4 Ireland; 5 School of Agriculture and Food Science, University College Dublin, Belfield, Dublin 4 Ireland

**Keywords:** *Serratia liquefaciens*, Carbon–fluorine bond, Enzyme, Dehalogenase, Fluoroacetate

## Abstract

**Abstract:**

The discovery of new enzymes that can hydrolyse the carbon–fluorine bond is important owing to the number of anthropogenic fluorinated compounds employed in a diverse range of applications and their recalcitrance in the environment. An enzyme from *Serratia liquefaciens* has been discovered that has dehalogenase, esterase and *N*-deformylase activities, a combination that has not previously been reported. The gene coding for the enzyme was expressed in *Escherichia coli* and the His-tagged protein purified via immobilised metal affinity chromatography. The enzyme defluorinated monofluoroethylglycine and fluoroacetate yielding homoserine and glycolate as defluorinated products, respectively. Esterase activity was established via *p*-nitrophenol acetate and *p*-nitrophenol butyrate hydrolysis; *N*-deformylation was measured by incubating the enzyme with *N*-formylmaleamic acid and detecting maleamic acid using gas chromatography-mass spectrometry. Based on sequence alignment, the catalytic triad was Ser-His-Asp, which is most unexpected as other fluoroacetate dehalogenases have a Asp-His-Asp triad. Site-directed mutagenesis of the presumed active site serine residue at position 101 to alanine resulted in the elimination of dehalogenating and esterase activities. In contrast, the S101D mutant had improved dehalogenase activity and diminished esterase activity compared to the wild type. Computational assessment of the presumptive catalytic triad (Ser-His-Asp) revealed a different orientation of His245 compared with the corresponding residue in fluoroacetate dehalogenase from *Burkholderia* sp. FA1. However, in the S101D mutant, the residues were much more closely aligned with those of the FA1 dehalogenase. Similar genes are present in other *Serratia* species and a number of other bacteria; thus, it is possible that other enzymes exist in the environment that have similar defluorinating activity. Such enzymes are a valuable resource for the development of biological methods for the remediation of organofluorine-polluted environments.

**Key points:**

• *SlDefH hydrolyses fluorinated substrates, esters and N-formylmaleamic acid*

• *An S101D mutant has improved dehalogenase and reduced esterase activity*

• *Enzymes with similar properties are likely to be present in the environment*

**Supplementary Information:**

The online version contains supplementary material available at 10.1007/s00253-026-13829-7.

## Introduction

Fluorinated compounds are widely used in a range of applications including agrochemicals, pharmaceuticals, refrigerants, propellants, cosmetic products, food packaging, non-stick cookware and stain-resistant fabrics (Harsanyi and Sandford 2015; Lewandowski et al. 2006). However, such compounds are often poorly degraded naturally and can accumulate in the environment leading to serious health effects in animals, including humans (Han et al. 2021). Of major concern is per- and polyfluorinated substances (PFAS), which are associated with a range of diseases including cancer, high cholesterol, high blood pressure and reduced immune response (Gluge et al. 2020).

In contrast to anthropogenic fluorinated compounds, naturally produced organofluorine compounds are rare, which is a consequence of the low bioavailability, low oxidation potential (−2.87 eV) and high heat of hydration (~ 120 kcal/mol) of the fluoride ion (O’Hagan and Deng 2015). The relative scarcity of naturally produced fluorinated compounds has delayed the evolution of specialised enzymes to degrade organofluorine compounds (Wackett 2024).

Nevertheless, microbial degradation of fluorinated aryl compounds, such as fluorobenzoate, fluorophenol and fluorobiphenyl, has been explored for decades. Owing to fluorine’s relatively small size, these compounds are catabolised along established pathways responsible for the degradation of aromatic compounds (Kiel and Engesser 2015; Moreira et al. 2018). The degree of biodegradation depends on the number and position of the fluorine atom(s) and yields fluoride ion and/or unmetabolisable ‘dead-end’ fluorometabolites. Fluorinated aliphatic compounds, for example, PFAS, are also partially biodegraded by microorganisms, but the pathways are not well understood (Dickman and Aga 2022; Lafond et al. 2023).

One class of microbial enzyme that specifically cleaves the carbon–fluorine bond is known, namely fluoroacetate dehalogenase. Enzymes belonging to this class have been identified in *Burkholderia*, *Pseudomonas*, *Rhodopseudomonas*, *Dechloromonas* and *Nostoc* species (Seong et al. 2019), amongst others, and the defluorinating mechanism has been established through crystallographic and mutational investigations (Jitsumori et al. 2009). Nucleophilic attack by an active site aspartate releases fluoride ion forming an ester intermediate, which is subsequently hydrolysed to form glycolate. There is also evidence that some fluoroacetate dehalogenases have broader substrate promiscuity and can degrade difluoroacetic acid (Khusnutdinova et al. 2023), thus may be further engineered to accept other multiply-fluorinated compounds and may eventually be applied to the biodegradation of PFAS.

We reported that a *Serratia liquefaciens* strain (IMD717), which was isolated from garden soil, degraded the fluorinated amino acid monofluoroethylglycine (MfeGly) yielding homoserine (Khan et al. 2023). Examination of the genome sequence revealed a putative dehalogenating enzyme that had a weak homology with known fluoroacetate dehalogenases and had a serine residue in place of the expected aspartate in the active site. At the time, no in vitro assessment of the enzyme activity had been conducted, and it was speculated that the enzyme was a broad range hydrolase that might also degrade fluorinated compounds.

In this paper, we report the cloning and expression of the enzyme in *E. coli* and demonstrate that it is indeed responsible for MfeGly degradation in *S. liquefaciens*. Furthermore, we examined the substrate scope of the enzyme and showed that the enzyme not only hydrolyses the C-F bond but is also an esterase and an *N*-deformylase. Such a combination of activities has not been observed previously. Comparison of the amino acid sequence with other available sequences suggests that those similar enzymes are widespread in the environment; thus, many more microorganisms than currently thought may be capable of hydrolysing fluorinated aliphatic compounds. These microorganisms and their enzymes may be applied directly to the degradation of certain organofluorine pollutants or serve as a starting point for enzyme engineering efforts to degrade more complex fluorinated compounds, such as PFAS.

## Materials and methods

### Chemicals

All chemicals were purchased from Merck (Arklow, Ireland), unless stated. The *N*-formylmaleamic acid was synthesised from maleic anhydride and formamidine acetate according to the method described by Behrman and Hillenbrand (2008). The crude product was employed for enzyme assay without purification. MfeGly was prepared as previously described (Khan et al. 2023).

### Expression and purification of dehalogenase

The protein and gene sequences of *Serratia liquefaciens* dehalogenase (SlDefH; accession number OP966820) were previously published in Khan et al. (2023). The SlDefH and mutant gene constructs with codon-optimised (OptimumGene™) sequences were synthesised by Genscript Biotech (Netherlands) and cloned into the pET28a vector using EcoRI and SalI restriction sites. *E. coli* XL10 and *E. coli* BL21 (DE3) served as the cloning and expression hosts, respectively. The QIAprep Spin Miniprep Kit and Ni–NTA Agarose beads were obtained from QIAGEN (Ireland).

The overexpression and purification of dehalogenases (wild type and mutants) followed the protocol outlined in Khan et al. (2019). Recombinant plasmids were purified from *E. coli* XL10 using the QIAprep Spin Miniprep Kit and transformed into competent *E. coli* BL21 (DE3). Transformants were spread on LB agar plate supplemented with 50 µg/mL kanamycin (kan). Colonies containing dehalogenase plasmids were cultured in 5 mL LB-kan broth as a primary culture. Subsequently, a 1% inoculum of this culture was added to 500 mL LB-kan broth in a 1-L flask, which was incubated at 37 °C with shaking at 180 rpm. When the OD_600_ reached 0.6 (2-h growth), gene expression was induced by adding 100 µM IPTG and incubating the cultures at 20 °C and 180 rpm for 24 h. The cells were harvested by centrifugation (9500×*g*, 4 °C, 10 min) and resuspended in 10 mL of 100 mM HEPES buffer (pH 7) with 200 mM NaCl and 0.5 mg/mL lysozyme. Soluble enzymes were obtained by sonication of the suspended cells (on ice) using a Sonics 130 W ultrasonic processor at 35% amplitude for a total of 20 min (5 s pulse on and 5 s pulse off) followed by centrifugation at 16,000×*g*, 4 °C, for 20 min. The supernatant was collected and the recombinant enzyme purified via Ni–NTA affinity chromatography. The pure enzyme was eluted with HEPES buffer supplemented with 25 mM (5 column volumes) and 250 mM (2 column volumes) imidazole, respectively. The enzyme purity was confirmed by SDS-PAGE.

To remove the imidazole from the purified dehalogenases, the solution was filtered through an Amicon Ultra-4 Centrifugal Filter with a 10 kDa MWCO (Millipore) by centrifugation at 5000×*g*, 4 °C, for 30 min. The retentate was diluted in HEPES (without imidazole) and the centrifugation step repeated three times. The protein concentrations were determined through the Bradford assay at 595 nm, using bovine serum albumin as a standard.

### Enzyme assay

SlDefH (0.1 mg/mL) was incubated with 1 mM of either monofluoroethylglycine (MfeGly), fluoroacetate (FAc), chloroacetate (ClAc), bromoacetate (BrAc) or *N*-formylmaleamic acid (N-FMA). Control experiments were conducted using boiled enzyme with the substrate in 100 mM HEPES buffer (pH 7) with 200 mM NaCl. To halt the enzyme reaction, the mixture was heated at 65 °C for 5 min.

Fluoride ion release was determined using an Orion Star A214 pH/ISE Meter equipped with an Orion™ Fluoride IonPlus Sure-Flow electrode following the method described in Khan et al. (2023). For fluorine-19 nuclear magnetic resonance spectroscopy (^19^F NMR), freeze-dried enzyme assay mixture was redissolved in D_2_O and analysed using a JEOL 400 MHz spectrometer.

To determine the hydrolysis products, gas chromatography-mass spectrometry (GC–MS) was employed. Samples of enzyme assay mixture were freeze-dried for 24–48 h, and the analytes derivatised by silylation by adding 100 µL of MSTFA and heating at 100 °C for 45 min. The liquid was transferred into a fresh 2-mL glass vial and analysed following the method described by Khan and Murphy (2022). Analysis was performed using a 7890B N Agilent GC system equipped with an HP-5MS capillary column (30 m × 0.25 mm × 0.33 µm) and a 5977 A mass-selective detector. Sample injection (1 µL) was carried out in split mode (5:1). The initial oven temperature was set at 90 °C for 3 min and then increased to 300 °C at a rate of 10 °C/min. The mass-selective detector operated in scan mode. To ensure no carry over between samples, injections of solvent blanks were routinely employed.

Glycolate concentrations were colorimetrically determined using the method developed by Takahashi (1972), in which samples of the enzyme assay were mixed with 0.01% 2,7-dihydroxynaphthalene in concentrated H_2_SO_4_. The resultant colour was measured at 540 nm using a Jenway 7315 spectrophotometer and the concentration of glycolate determined by comparison to a standard curve (Figure S1).

### Enzyme characterisation and kinetic analysis

Esterase and dehalogenase activities of SlDefH were assessed spectrophotometrically, using *p*-nitrophenol acetate (p-NPA), *p*-nitrophenol butyrate and FAc as substrates, on a Jenway 7315 spectrophotometer. For esterase activity, the liberated *p*-nitrophenol (p-NP) was measured spectrophotometrically at 410 nm (ɛ = 17,500 M^−1^cm^−1^). Dehalogenase activity was determined by measuring fluoride ion following the method described by Bygd et al. (2022). A standard curve of the fluoride ion concentration (5–100 µM) plotted against the ratio of absorbance at 620 and 530 nm was employed (Figure S2). There was no interference from any of the assay components.

One enzyme unit (U) was defined as the esterase activity releasing 1 µmol equivalent of p-NP per mL per minute and dehalogenase activity releasing 1 µmol equivalent of F^−^ ion per mL per minute under standard assay conditions.

The kinetic parameters specific activity, K_m_ value, turnover number (k_cat_) and catalytic efficiency (k_cat_/K_m_) were calculated for both esterase and dehalogenase activities. To establish the optimum temperature and pH for the enzyme, SlDefH was individually incubated with p-NPA (for 30 min) and FAc (for 1 h) at specific temperatures of 20, 25, 30, 35, 40, 45, 50 and 55 °C and pH values of 3.0, 4.0, 5.0, 6.0, 6.5, 7.0, 7.5, 8.0, 9.0 and 10.0. The pH was maintained using the following buffer systems, all prepared at a final concentration of 100 mM: citrate buffer (pH 3.0), acetate buffer (pH 4.0–6.0), HEPES buffer (pH 6.5–8.0), Tris–HCl buffer (pH 9.0) and glycine–NaOH buffer (pH 10.0). The effect of some metal ions on enzyme activity (Mg^2+^, Ca^2+^, Co^2+^ and Fe^3+^) was determined by conducting the assays in the presence of 5 mM of the metal or EDTA.

### Phylogenetic analysis

A phylogenetic tree was assembled using 18 protein sequences in total, including the dehalogenase from *Serratia liquefaciens* IMD717 and 17 homologous proteins from other *Serratia* species. These sequences included α/β-hydrolases, tropinesterases and *N*-formylmaleamate deformylases with ≥ 80% amino-acid sequence similarity, identified through BLASTp searches against the NCBI non-redundant protein database. The accession numbers of all sequences used in the analysis are provided in Fig. 3 to ensure reproducibility. Multiple sequence alignment was performed using the Clustal Omega algorithm (https://www.ebi.ac.uk/Tools/msa/clustalo/).

The aligned sequences were used to construct a phylogenetic tree using the neighbour-joining (NJ) method, and branch robustness was evaluated by bootstrap analysis with 1000 replicates. The resulting tree was visualised and annotated using the Interactive Tree Of Life (iTOL) v6 online tool (https://itol.embl.de/).

### In silico mutagenesis and structural alignment

To generate the 3D protein structures, the sequences of SlDefH and the mutant S101D were submitted to the Phyre2 online server (https://www.sbg.bio.ic.ac.uk/phyre2). This server modelled both variants using the template of fluoroacetate dehalogenase from *Burkholderia* sp. FA1 (PDB ID: 1Y37) with a confidence level of 100%.

To assess the structural changes in the active site induced by the S101D mutation, the modelled structures were then aligned with the 1Y37 PDB structure and visualised using the PyMOL 2.5. The distances between the aligned catalytic residues of SlDefH wild type, mutant and 1Y37 were measured.

## Results

### *S. liquefaciens *IMD717 has a novel defluorinating enzyme

**Fig. 1:**
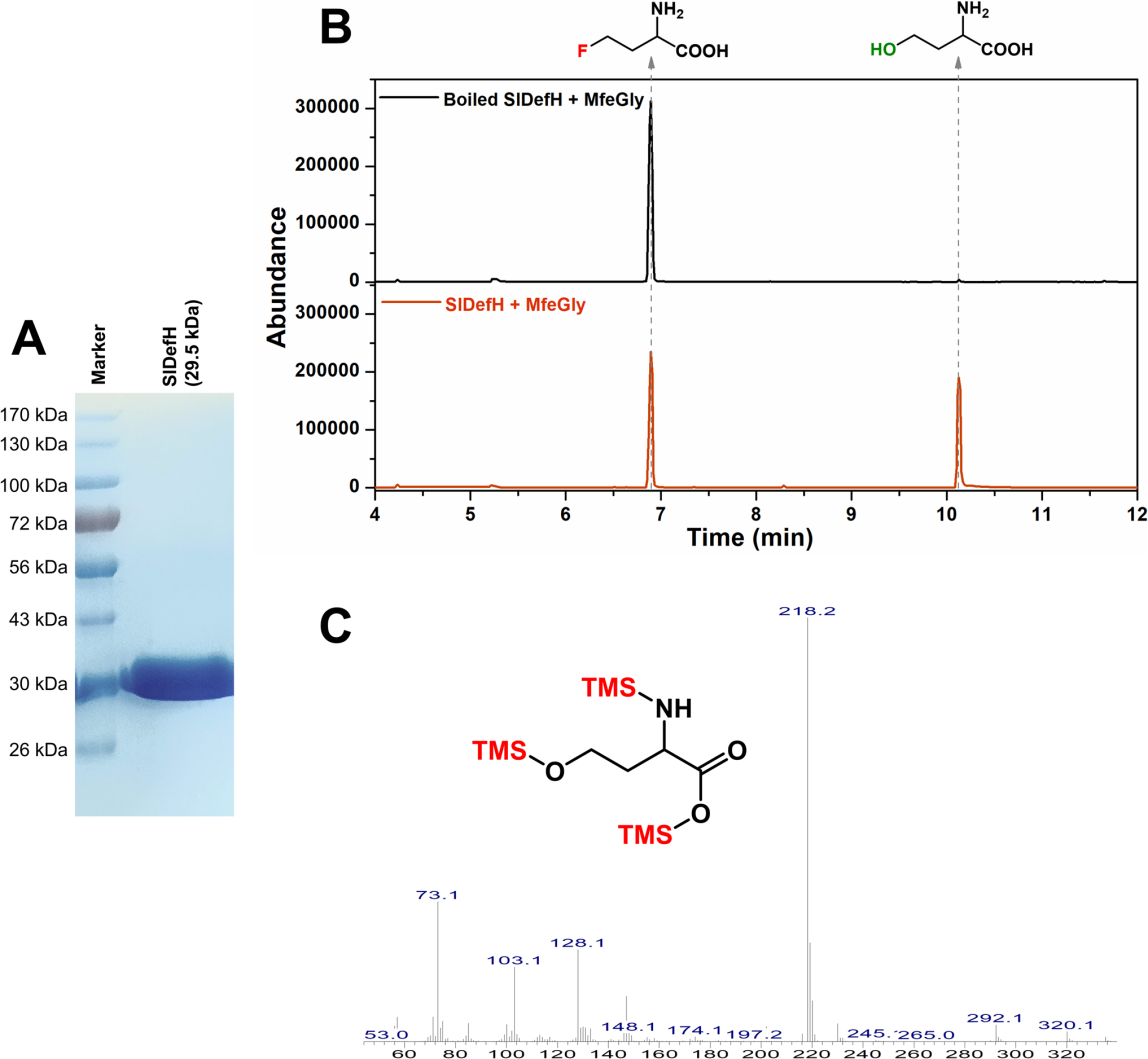
The proposed dehalogenase (SlDefH) from *S. liquefaciens* IMD717 was expressed in *E. coli* and the histidine-tagged protein was purified from cell free extracts. Purity was established by SDS-PAGE (**A**) and the protein had the expected mass (29.5 kDa) based on the primary amino acid sequence. Activity was assessed by incubation with MfeGly yielding a new peak that was detected by GC–MS analysis (**B**), using boiled enzyme as a control. The identity of the new peak at 10.1 min was confirmed as homoserine via comparison of the mass spectrum (**C**) with that of an authentic standard (Figure S3)

In our previous paper (Khan et al. 2023), we identified a gene that had a translated sequence with some similarities to known fluoroacetate dehalogenases, albeit with an overall low homology. However, no assay with a purified enzyme was reported at the time. To confirm that the *S. liquefaciens* enzyme had defluorinating activity, the gene was cloned and expressed in *E. coli* and the resultant protein purified by immobilised metal affinity chromatography (Fig. 1A). The enzyme was initially assayed with MfeGly as the substrate, as this was the compound used in our previous study that led to the isolation of the bacterium. A new peak was detected by GC–MS that was not present in a control experiment using boiled enzyme (Fig. 1B). This peak was confirmed as the silylated derivative of homoserine by comparing its retention time and mass spectrum with that of an authentic standard. This was the same product that was found by Khan et al. (2023) when the same substrate was incubated with cell free extracts of *S. liquefaciens* IMD717. The presence of fluoride ion was confirmed ion selective electrode, which determined 0.25 mM fluoride ion after MfeGly was incubated for 2 h incubation with the purified enzyme. There was no fluoride ion detected in the boiled enzyme control experiment. Thus, the enzyme had the expected defluorinating activity and was renamed *S. liquefaciens* defluorinating hydrolase (SlDefH) for the purposes of this study.

**Fig. 2:**
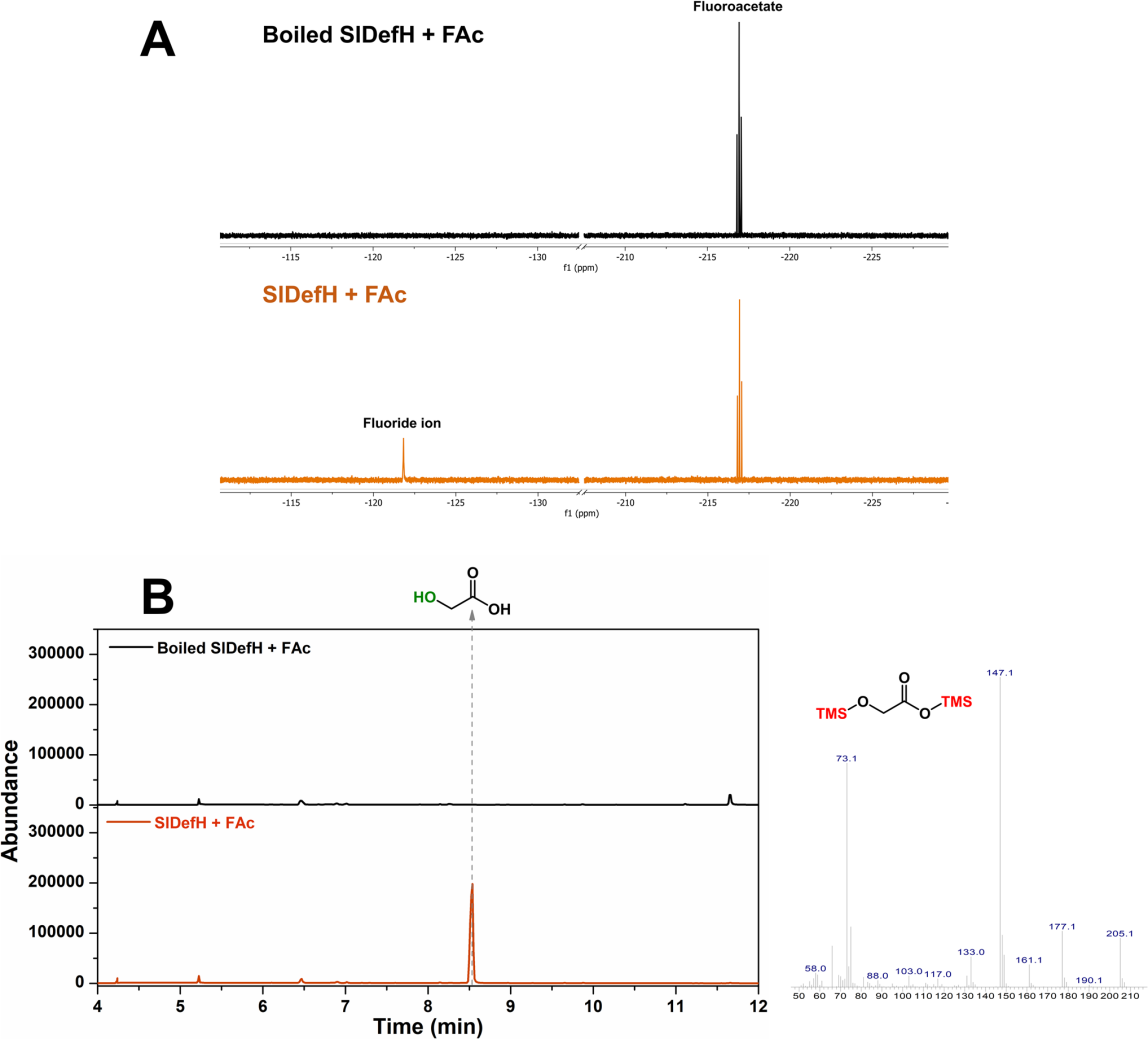
Defluorination of fluoroacetate by SlDefH. ^19^F NMR revealed fluoride ion as a singlet at δ −122 ppm, which is not present when a boiled enzyme is used (**A**); fluoroacetate appears as a triplet at δ −216 ppm. The defluorinated product glycolate was determined by GC–MS (**B**) and was also absent in a boiled enzyme control experiment. The mass spectrum of the silylated product is also shown, which is identical to that of an authentic standard of silylated glycolate

The enzyme was also assayed for the dehalogenation of haloacetates. Incubation of fluoroacetate with the enzyme resulted in release of fluoride ion that could be detected using a fluoride ion selective electrode and ^19^F NMR. The latter revealed the presence of fluoride ion when the substrate was incubated with active enzyme, but not in a boiled enzyme control experiment (Fig. 2A). Also, the defluorination product of fluoroacetate was expected to be glycolate and this compound was detected by GC–MS analysis of the enzyme assay mixture following silylation. Figure 2B shows the presence of a new peak eluting at 8.5 min that was not present in the control experiment with boiled enzyme and had the expected mass spectrum of glycolate. Colorimetric determination of glycolate revealed 0.265 mM glycolate after 2-h incubation of fluoroacetate with the enzyme, which was almost the same as the fluoride ion concentration determined using the ion selective electrode method (0.23 mM) confirming the expected stoichiometry. The enzyme also dehalogenated chloroacetate and bromoacetate yielding glycolate, which was detected by GC–MS (Figure S4) and colorimetrically. The halide ions from these substrates were not determined but it is assumed that the same dehalogenation reaction occurred. Other fluorinated compounds such as trifluoroacetate, difluoroethylglycine, perfluorooctanoic acid (PFOA) and 6:2 fluorotelomer alcohol (6:2 FTOH) were also assessed as substrates but no defluorination was detected, suggesting the enzyme has a narrow specificity for fluorinated compounds.

### The *S. liquefaciens *dehalogenase is also an esterase and *N*-deformylase

**Fig. 3:**
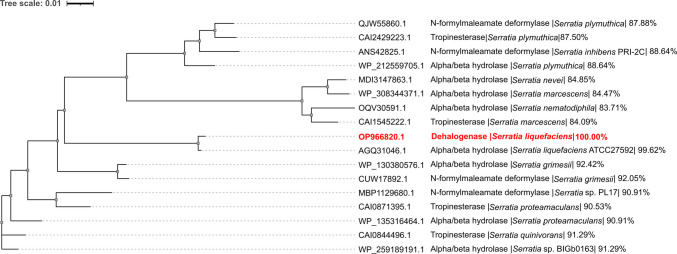
Phylogenetic tree showing the relationship between the dehalogenase activity detected in *S. liquefaciens* IMD717 (coloured red) and a selection of other hydrolases in *Serratia* spp. The percentage similarity is also shown

It was previously reported that the SlDeFH enzyme had a low similarity with known fluoroacetate dehalogenases (Khan et al. 2023) and a BLASTp analysis of the translated protein revealed that the top 100 most closely related proteins based on their E-values were αβ fold hydrolases from a number *Serratia* species with percentage identities of at least 85% (Table S1). This suggests that other *Serratia* spp. may have enzymes with similar defluorinating activities. The phylogenetic tree in Fig. 3 shows the relationship between SlDefH and a selection of similar proteins identified by pBLAST some of whom were outside the closest matches but had a proposed function. Some of the enzymes that also had a relatively high sequence similarity to SlDefH were further identified as tropinesterase and *N*-formylmaleamate deformylase. The former functions in the metabolism of alkaloids (Long et al. 1993) and the latter is an enzyme that converts *N*-formylmaleamate to formate as part of the nicotine degradation pathway (Wu et al. 2014).

Based on the BLASTp analysis, we investigated if the enzyme hydrolysed other (non-halogenated) substrates by conducting in vitro assays of the purified enzyme for esterase and *N*-deformylase activities.

**Fig. 4:**
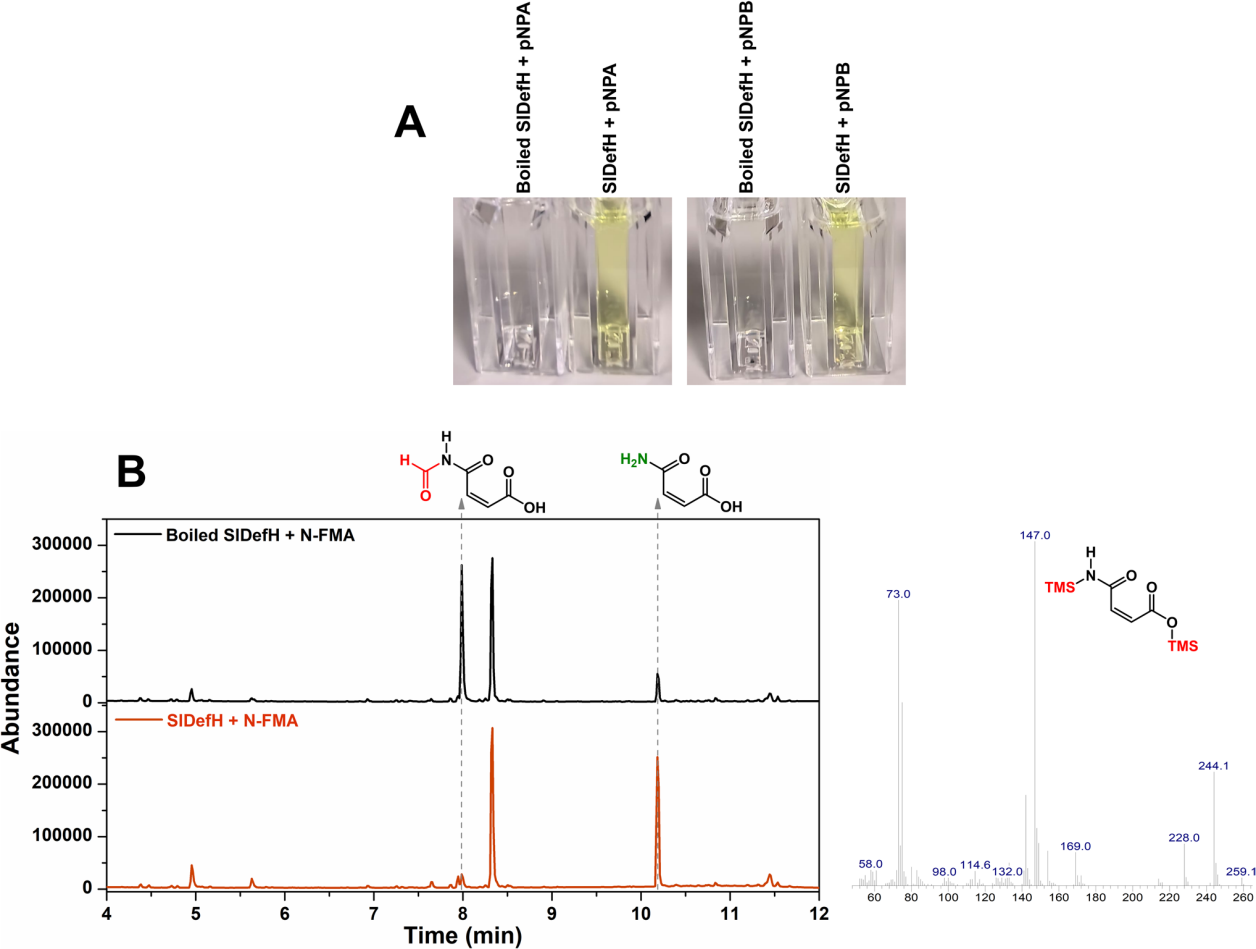
The *S. liquefaciens* enzyme has a promiscuous functionality and can catalyse hydrolysis of, pNPA and pNPB yielding nitrophenol (**A**) and *N*-formylmaleamate yielding maleamate (**B**). There is a small amount of contaminating maleamate in the boiled enzyme control experiment, which is present as an impurity from the synthesis; however, it is clear that the amount present after incubation with active enzyme is dramatically increased

Esterase activity is commonly assayed using pNPA or pNPB as a substrate, as the *p*-nitrophenol product can be readily observed from the yellow colour. Figure 4A shows the colour difference in assays conducted with pNPA and pNPB incubated with SlDefH and the boiled enzyme, demonstrating esterase activity. *N*-deformylase activity was determined by using GC–MS to detect the presence of maleamic acid, which is formed upon hydrolysis of *N*-formylmaleamic acid. Figure 4B shows the disappearance of the substrate, which elutes after 8 min, and a dramatic increase in the peak at 10.2 min, which has the expected mass spectrum of maleamic acid (Figure S5). Thus, SlDefH has defluorinase, esterase and *N*-deformylase activity, confirming it as a broad range hydrolase.

Optimum pH and temperature ranges for the esterase and dehalogenase activities were established and showed that the enzyme was mesophilic for both activities with a temperature optimum of 35 °C (Figure S6). The pH optimum differed for the dehalogenase (7) and esterase (8) activities, although both are not outside those typically reported. Addition of EDTA to the enzyme assay did not impact either dehalogenase or esterase activity, suggesting that the enzyme was not a metalloprotein. None of the metal ions tested markedly affected the activity (Table S2).

### Mutation of active the site serine residue enhances defluorinating activity

Our previous report (Khan et al. 2023) highlighted a difference in the key active site residue at position 101 in the sequence alignment of *Serratia liquefaciens* dehalogenase (SlDefH) compared to three known fluoroacetate dehalogenases from *Burkholderia* sp. (protein ID Q1JU72), *Rhodopseudomonas palustris* (protein ID Q6NAM1) and *Delftia acidovorans* (protein ID Q01398). All the previously reported dehalogenases had an aspartate at the active site in this aligned position, whereas in SlDefH, this residue is serine. To investigate the importance of Ser101 in SlDefH, an S101A mutant was expressed in *E. coli*, purified and assayed for dehalogenase and esterase activity as previously described. Dehalogenase and esterase activities were eliminated in the mutant, confirming that this residue is catalytically important. However, it is unlikely to be the nucleophile in the dehalogenation rection as it would result in the formation of a stable ether instead of the ester that would be formed with aspartate. It was also hypothesised that the dehalogenating activity of SlDefH might be improved in an S101D mutant, so the corresponding gene was synthesised and expressed in *E. coli*. The resultant purified enzyme was assayed with fluoroacetate as before and the kinetic properties of the mutant and wild-type enzymes were compared (Table 1; Figure S7), showing that for the mutant, there was a decrease in Km and increase in kcat, ultimately leading to a 65% improvement in catalytic efficiency. Conversely, the esterase efficiency was approx. 65% lower in the mutant compared to the wild type.

**Table 1:** Kinetic parameters of SlDefH wild type and S101D mutant catalysing dehalogenase and esterase activities. The values are means ± standard deviation

Parameter	SlDefH	SlDefH-S101D
Dehalogenase activity		
Specific activity or Vmax (U mg^−1^)	33.3 ± 1.2	44.3 ± 1.3
Km (mM)	2.9 ± 0.4	2.2 ± 0.3
Turnover number, kcat (s^−1^)	332.6 ± 12.4	441.8 ± 12.3
Catalytic efficiency kcat/Km (s^−1^ mM^−1^)	114.0 ± 12.3	190.1 ± 12.1
Esterase activity		
Specific activity or Vmax (U mg^−1^)	131.3 ± 12.2	97.8 ± 11.6
Km (mM)	1.0 ± 0.2	1.6 ± 0.3
Turnover number, kcat (s^−1^)	1309.9 ± 122.1	976.3 ± 115.4
Catalytic efficiency kcat/Km (s^−1^ mM^−1^)	1395.3 ± 188.0	628.2 ± 76.7

**Fig. 5:**
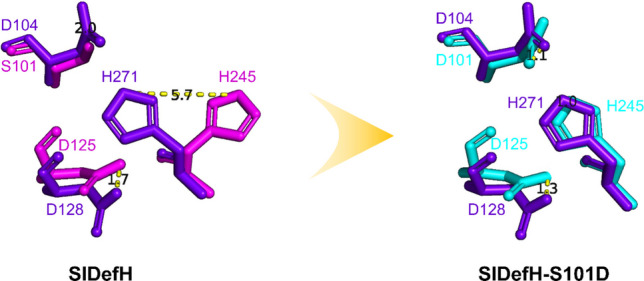
Superimposition of the catalytic triad in the active site of SlDefH (pink) and the S101D mutant (cyan) with that of FAc dehalogenase from *Burkholderia* (purple)

By using the Phyre2 server, it was possible to compare the orientation of the Ser-Asp-His catalytic triad in SlDefH with the corresponding residues in fluoroacetate dehalogenase from *Burkholderia.* As can be seen in Fig. 5, there is an obvious difference in the orientation in the histidine residue in the two enzymes. Notably, the histidine is closer to the aspartate nucleophile in fluoroacetate dehalogenase which might more readily facilitate the hydrolysis of the ester intermediate in the catalytic cycle. Curiously, the model indicates this difference is eliminated if Ser101 in SlDefH is replaced with an aspartic acid residue. When the active site Asp104 was exchanged for serine in the *Burkholderia* enzyme, there was no change in the orientation of histidine (not shown).

## Discussion

A novel broad range hydrolase, SlDefH, has been identified in *S. liquefaciens* that has esterase, *N*-deformylase and dehalogenating activities. So far as we can tell, no enzyme with this combination of activities has been previously reported. We had proposed the putative protein to be responsible for the defluorinating activity observed in this bacterium, based on sequence comparisons with known fluoroacetate dehalogenases, but had not conducted any in vitro experiments (Khan et al. 2023). The sequencing analysis revealed that there was a low overall homology with the known defluorinating enzymes, but the key residues of these enzymes could be identified, specifically Asp 125, His 145 and Trp 142.

One crucial difference of SlDefH compared to fluoroacetate dehalogenases was the presence of serine in place of the conserved nucleophilic aspartate residue. Despite this difference, the specific activity of the enzyme measured with fluoroacetate as the substrate is comparable to fluoroacetate dehalogenases in *Burkholderia* (Kurihara et al. 2003) and *Moraxella* sp. B (Lui et al. 1998). The (halogenated) substrate range of SlDeFH is limited to monohalogenated compounds, which is also similar to the fluoroacetate dehalogenases in *Burkholderia* and *Moraxella*, although it is narrower than the fluoroacetate dehalogenases from bacteria such as *Dechloromonas aromatica* and *Rhodopseudomonas palustris* that can defluorinate difluoroacetate (Khusnutdinova et al. 2023).

One hybrid esterase-dehalogenase, REBr, has previously been identified that was obtained via mining a marine metagenomic library, which also has a serine residue rather than aspartate in the presumed catalytic triad (Beloqui et al. 2010). Using radioactively labelled substrates these researchers demonstrated that the active site serine was important for the esterase activity, but identified a glutamic acid residue that was also present in the active site, which played the role of nucleophile in dehalogenating reactions. In the experiments reported in the present paper, an S101A mutant did not have measurable dehalogenating activity, suggesting a role for the active site serine in the dehalogenation reaction. Beloqui et al. also found that mutating the active site serine had a dramatic effect on the dehalogenating activity of REBr but reasoned that this was due to its importance in substrate binding; it is possible that the S101 plays a similar role in SlDefH, and this will be a focus of future investigations.

Promiscuity is common in α/β hydrolases and is thought to be important for the evolution of new enzymatic activities (Rauwerdink and Kazlauskas 2015); however, examples of hydrolases that catalyse dehalogenation in addition to their main activity are very rare. Aside from REBr and SlDefH, only one other is known, which is the epoxide hydrolase CorEH that can dehalogenate haloalkanes (Schuiten et al. 2021). In this example, the specific activity of dehalogenation was 5000 times lower than that of the epoxide hydrolase activity.

The SlDefH enzyme has a high homology with many available protein sequences, which have the same putative key residues that are thought to be important for the dehalogenating activity (Figure S8); thus, it might be that there are other similar hydrolases in the environment that catalyse defluorination. Enzymes that can hydrolytically cleave the carbon–fluorine bond are important components of the ‘molecular toolkit’ for the removal of PFAS (Hu and Scott 2024). The enzyme identified here was unable to degrade difluoro- or polyfluorinated compounds; nevertheless, it could be employed for enzyme engineering campaigns to broaden its substrate scope beyond fluoroacetate and monofluoroethylglycine. Such engineering efforts employing the fluoroacetate dehalogenase from *Delftia acidovorans* have yielded variants that had altered substrate profiles for 2-fluoropropionate and 2,2-difluoroacetate (Jansen et al. 2026).

The current work provides an additional insight into hydrolase promiscuity, highlights the possibility of many other enzymes with the potential for defluorinase activity in the environment and provides a new candidate for enzyme engineering to enable biodegradation of hazardous fluorinated compounds. Future work will focus on determining if similar enzymes in other bacteria have comparable activity and how they might be employed in the bioremediation of organofluorine pollution.

## Supplementary Information

Below is the link to the electronic supplementary material.

ESM 1 DOCX (2.25 MB)

## Data Availability

The datasets used and analysed during the current study are available from the corresponding author on reasonable request.
