# Supplementary material for: A hydrolase from Serratia liquefaciens IMD717 with esterase, dehalogenase and N-deformylase activities
Source: Appl Microbiol Biotechnol. 2026 Apr 20;110(1):167. doi: 10.1007/s00253-026-13829-7 (PMC13230308; doi:10.1007/s00253-026-13829-7)

**A hydrolase from *Serratia liquefaciens* IMD717 with esterase, dehalogenase and *N*-deformylase activities**

Mohd Faheem Khan ^a, e^, Periklis Karamanis ^b^, Beate Koksch ^c^ and Cormac D. Murphy ^a, d*^

^a^ School of Biomolecular and Biomedical Science, University College Dublin, Belfield, Dublin 4, Ireland

^b^ School of Chemistry, University College Dublin, Belfield, Dublin 4, Ireland

^c^ Institute of Chemistry and Biochemistry, Freie Universität Berlin, Arnimallee 20, 14195 Berlin, Germany

^d^ UCD Conway Institute, University College Dublin, Belfield, Dublin 4, Ireland

^e^ School of Agriculture and Food Science, University College Dublin, Belfield, Dublin 4, Ireland

*Corresponding author: [cormac.d.murphy@ucd.ie](mailto:cormac.d.murphy@ucd.ie)

**Supplemental Information**

**Synthesis of N-formylmaleamic acid**

Formamidine acetate (1.0 g, 0.01 mol) was added to a round-bottom flask and dissolved in acetone/H_2_O (95:5, 50 mL). Maleic anhydride (0.98 g, 0.01 mol) was added with stirring and the solution became clear. After 15 minutes, the solvents were removed *in vacuo*, yielding 0.9 g of the crude mixture containing N-formylmaleamic acid (**1**). The product was confirmed by GC-MS after silylation.

Table S1. BLASTp comparison of SlFacD with proteins in GenBank

| Description | Scientific Name | Max Score | Total Score | Query Cover | E value | Per. ident | Acc. Len | Accession |
| --- | --- | --- | --- | --- | --- | --- | --- | --- |
| MULTISPECIES: alpha/beta fold hydrolase [Serratia] | Serratia | 547 | 547 | 100% | 0 | 100 | 264 | [WP_116690531.1](https://www.ncbi.nlm.nih.gov/protein/WP_116690531.1?report=genbank&log$=prottop&blast_rank=1&RID=KD2R5136014) |
| alpha/beta fold hydrolase [Serratia liquefaciens] | Serratia liquefaciens | 546 | 546 | 100% | 0 | 99.62 | 264 | [WP_187962835.1](https://www.ncbi.nlm.nih.gov/protein/WP_187962835.1?report=genbank&log$=prottop&blast_rank=2&RID=KD2R5136014) |
| alpha/beta fold hydrolase [Serratia liquefaciens] | Serratia liquefaciens | 546 | 546 | 100% | 0 | 99.62 | 264 | [WP_115058883.1](https://www.ncbi.nlm.nih.gov/protein/WP_115058883.1?report=genbank&log$=prottop&blast_rank=3&RID=KD2R5136014) |
| alpha/beta fold hydrolase [Serratia liquefaciens] | Serratia liquefaciens | 546 | 546 | 100% | 0 | 99.62 | 264 | [WP_046373626.1](https://www.ncbi.nlm.nih.gov/protein/WP_046373626.1?report=genbank&log$=prottop&blast_rank=4&RID=KD2R5136014) |
| MULTISPECIES: alpha/beta fold hydrolase [Serratia] | Serratia | 545 | 545 | 100% | 0 | 99.62 | 264 | [WP_020826784.1](https://www.ncbi.nlm.nih.gov/protein/WP_020826784.1?report=genbank&log$=prottop&blast_rank=5&RID=KD2R5136014) |
| alpha/beta fold hydrolase [Serratia liquefaciens] | Serratia liquefaciens | 545 | 545 | 100% | 0 | 99.62 | 264 | [WP_130015694.1](https://www.ncbi.nlm.nih.gov/protein/WP_130015694.1?report=genbank&log$=prottop&blast_rank=6&RID=KD2R5136014) |
| alpha/beta fold hydrolase [Serratia liquefaciens] | Serratia liquefaciens | 545 | 545 | 100% | 0 | 99.62 | 264 | [WP_129940556.1](https://www.ncbi.nlm.nih.gov/protein/WP_129940556.1?report=genbank&log$=prottop&blast_rank=7&RID=KD2R5136014) |
| alpha/beta hydrolase [Serratia liquefaciens] | Serratia liquefaciens | 545 | 545 | 100% | 0 | 99.62 | 264 | [HBK4767004.1](https://www.ncbi.nlm.nih.gov/protein/HBK4767004.1?report=genbank&log$=prottop&blast_rank=8&RID=KD2R5136014) |
| alpha/beta hydrolase [Serratia liquefaciens] | Serratia liquefaciens | 545 | 545 | 100% | 0 | 99.24 | 264 | [WP_260628787.1](https://www.ncbi.nlm.nih.gov/protein/WP_260628787.1?report=genbank&log$=prottop&blast_rank=9&RID=KD2R5136014) |
| alpha/beta hydrolase [Serratia liquefaciens] | Serratia liquefaciens | 545 | 545 | 100% | 0 | 99.62 | 264 | [HEJ7889288.1](https://www.ncbi.nlm.nih.gov/protein/HEJ7889288.1?report=genbank&log$=prottop&blast_rank=10&RID=KD2R5136014) |
| MULTISPECIES: alpha/beta fold hydrolase [Serratia] | Serratia | 544 | 544 | 100% | 0 | 99.24 | 264 | [WP_122079251.1](https://www.ncbi.nlm.nih.gov/protein/WP_122079251.1?report=genbank&log$=prottop&blast_rank=11&RID=KD2R5136014) |
| alpha/beta hydrolase [Serratia liquefaciens] | Serratia liquefaciens | 544 | 544 | 100% | 0 | 99.24 | 264 | [WP_413510186.1](https://www.ncbi.nlm.nih.gov/protein/WP_413510186.1?report=genbank&log$=prottop&blast_rank=12&RID=KD2R5136014) |
| alpha/beta hydrolase [Serratia liquefaciens] | Serratia liquefaciens | 544 | 544 | 100% | 0 | 99.24 | 264 | [HCT7984277.1](https://www.ncbi.nlm.nih.gov/protein/HCT7984277.1?report=genbank&log$=prottop&blast_rank=13&RID=KD2R5136014) |
| alpha/beta fold hydrolase [Serratia liquefaciens] | Serratia liquefaciens | 543 | 543 | 100% | 0 | 98.86 | 264 | [EPA9109861.1](https://www.ncbi.nlm.nih.gov/protein/EPA9109861.1?report=genbank&log$=prottop&blast_rank=14&RID=KD2R5136014) |
| alpha/beta hydrolase [Serratia liquefaciens] | Serratia liquefaciens | 543 | 543 | 100% | 0 | 99.24 | 264 | [HBL7238613.1](https://www.ncbi.nlm.nih.gov/protein/HBL7238613.1?report=genbank&log$=prottop&blast_rank=15&RID=KD2R5136014) |
| MULTISPECIES: alpha/beta fold hydrolase [Serratia] | Serratia | 543 | 543 | 100% | 0 | 99.62 | 264 | [WP_198639606.1](https://www.ncbi.nlm.nih.gov/protein/WP_198639606.1?report=genbank&log$=prottop&blast_rank=16&RID=KD2R5136014) |
| alpha/beta fold hydrolase [Serratia liquefaciens] | Serratia liquefaciens | 543 | 543 | 100% | 0 | 99.24 | 264 | [WP_261157021.1](https://www.ncbi.nlm.nih.gov/protein/WP_261157021.1?report=genbank&log$=prottop&blast_rank=17&RID=KD2R5136014) |
| alpha/beta hydrolase [Serratia liquefaciens] | Serratia liquefaciens | 543 | 543 | 100% | 0 | 99.24 | 264 | [HBL6731233.1](https://www.ncbi.nlm.nih.gov/protein/HBL6731233.1?report=genbank&log$=prottop&blast_rank=18&RID=KD2R5136014) |
| alpha/beta fold hydrolase [Serratia liquefaciens] | Serratia liquefaciens | 541 | 541 | 100% | 0 | 98.86 | 264 | [WP_404779855.1](https://www.ncbi.nlm.nih.gov/protein/WP_404779855.1?report=genbank&log$=prottop&blast_rank=19&RID=KD2R5136014) |
| alpha/beta fold hydrolase [Serratia quinivorans] | Serratia quinivorans | 509 | 509 | 100% | 0 | 91.29 | 264 | [WP_261094918.1](https://www.ncbi.nlm.nih.gov/protein/WP_261094918.1?report=genbank&log$=prottop&blast_rank=20&RID=KD2R5136014) |
| alpha/beta fold hydrolase [Serratia quinivorans] | Serratia quinivorans | 507 | 507 | 100% | 2.00E-180 | 91.29 | 264 | [WP_261430406.1](https://www.ncbi.nlm.nih.gov/protein/WP_261430406.1?report=genbank&log$=prottop&blast_rank=21&RID=KD2R5136014) |
| alpha/beta fold hydrolase [Serratia proteamaculans] | Serratia proteamaculans | 503 | 503 | 100% | 5.00E-179 | 90.91 | 264 | [WP_135316464.1](https://www.ncbi.nlm.nih.gov/protein/WP_135316464.1?report=genbank&log$=prottop&blast_rank=22&RID=KD2R5136014) |
| alpha/beta fold hydrolase [Serratia plymuthica] | Serratia plymuthica | 497 | 497 | 100% | 2.00E-176 | 88.64 | 264 | [WP_212559705.1](https://www.ncbi.nlm.nih.gov/protein/WP_212559705.1?report=genbank&log$=prottop&blast_rank=23&RID=KD2R5136014) |
| alpha/beta fold hydrolase [Serratia sp. root2] | Serratia sp. root2 | 497 | 497 | 100% | 2.00E-176 | 89.39 | 264 | [WP_311992772.1](https://www.ncbi.nlm.nih.gov/protein/WP_311992772.1?report=genbank&log$=prottop&blast_rank=24&RID=KD2R5136014) |
| alpha/beta fold hydrolase [Serratia inhibens] | Serratia inhibens | 496 | 496 | 100% | 3.00E-176 | 88.64 | 264 | [WP_065506278.1](https://www.ncbi.nlm.nih.gov/protein/WP_065506278.1?report=genbank&log$=prottop&blast_rank=25&RID=KD2R5136014) |
| alpha/beta fold hydrolase [Serratia plymuthica] | Serratia plymuthica | 496 | 496 | 100% | 6.00E-176 | 89.39 | 264 | [WP_325986503.1](https://www.ncbi.nlm.nih.gov/protein/WP_325986503.1?report=genbank&log$=prottop&blast_rank=26&RID=KD2R5136014) |
| alpha/beta fold hydrolase [Serratia plymuthica] | Serratia plymuthica | 496 | 496 | 100% | 6.00E-176 | 89.39 | 264 | [WP_122290117.1](https://www.ncbi.nlm.nih.gov/protein/WP_122290117.1?report=genbank&log$=prottop&blast_rank=27&RID=KD2R5136014) |
| alpha/beta fold hydrolase [Serratia plymuthica] | Serratia plymuthica | 496 | 496 | 100% | 6.00E-176 | 89.39 | 264 | [WP_004944096.1](https://www.ncbi.nlm.nih.gov/protein/WP_004944096.1?report=genbank&log$=prottop&blast_rank=28&RID=KD2R5136014) |
| alpha/beta fold hydrolase [Serratia plymuthica] | Serratia plymuthica | 494 | 494 | 100% | 3.00E-175 | 89.02 | 264 | [WP_208905058.1](https://www.ncbi.nlm.nih.gov/protein/WP_208905058.1?report=genbank&log$=prottop&blast_rank=29&RID=KD2R5136014) |
| alpha/beta fold hydrolase [Serratia sp. 22264] | Serratia sp. 22264 | 494 | 494 | 100% | 3.00E-175 | 88.64 | 264 | [WP_440832658.1](https://www.ncbi.nlm.nih.gov/protein/WP_440832658.1?report=genbank&log$=prottop&blast_rank=30&RID=KD2R5136014) |
| alpha/beta fold hydrolase [Serratia plymuthica] | Serratia plymuthica | 493 | 493 | 100% | 6.00E-175 | 89.02 | 264 | [WP_409442564.1](https://www.ncbi.nlm.nih.gov/protein/WP_409442564.1?report=genbank&log$=prottop&blast_rank=31&RID=KD2R5136014) |
| alpha/beta fold hydrolase [Serratia quinivorans] | Serratia quinivorans | 493 | 493 | 100% | 1.00E-174 | 92.42 | 264 | [WP_442231876.1](https://www.ncbi.nlm.nih.gov/protein/WP_442231876.1?report=genbank&log$=prottop&blast_rank=32&RID=KD2R5136014) |
| alpha/beta hydrolase [Serratia plymuthica] | Serratia plymuthica | 492 | 492 | 100% | 2.00E-174 | 87.88 | 264 | [BFO84823.1](https://www.ncbi.nlm.nih.gov/protein/BFO84823.1?report=genbank&log$=prottop&blast_rank=33&RID=KD2R5136014) |
| alpha/beta hydrolase [Serratia plymuthica] | Serratia plymuthica | 492 | 492 | 100% | 2.00E-174 | 87.88 | 264 | [WP_062792432.1](https://www.ncbi.nlm.nih.gov/protein/WP_062792432.1?report=genbank&log$=prottop&blast_rank=34&RID=KD2R5136014) |
| alpha/beta hydrolase [Serratia sp. TSA_198.1] | Serratia sp. TSA_198.1 | 491 | 491 | 100% | 3.00E-174 | 88.26 | 264 | [WP_445583778.1](https://www.ncbi.nlm.nih.gov/protein/WP_445583778.1?report=genbank&log$=prottop&blast_rank=35&RID=KD2R5136014) |
| alpha/beta fold hydrolase [Serratia grimesii] | Serratia grimesii | 491 | 491 | 100% | 4.00E-174 | 92.42 | 264 | [WP_130380576.1](https://www.ncbi.nlm.nih.gov/protein/WP_130380576.1?report=genbank&log$=prottop&blast_rank=36&RID=KD2R5136014) |
| alpha/beta hydrolase [Serratia plymuthica] | Serratia plymuthica | 491 | 491 | 100% | 4.00E-174 | 88.26 | 264 | [WP_073439503.1](https://www.ncbi.nlm.nih.gov/protein/WP_073439503.1?report=genbank&log$=prottop&blast_rank=37&RID=KD2R5136014) |
| alpha/beta fold hydrolase [Serratia grimesii] | Serratia grimesii | 490 | 490 | 100% | 8.00E-174 | 92.42 | 264 | [WP_061806151.1](https://www.ncbi.nlm.nih.gov/protein/WP_061806151.1?report=genbank&log$=prottop&blast_rank=38&RID=KD2R5136014) |
| alpha/beta fold hydrolase [Serratia plymuthica] | Serratia plymuthica | 490 | 490 | 100% | 1.00E-173 | 87.88 | 264 | [WP_006325071.1](https://www.ncbi.nlm.nih.gov/protein/WP_006325071.1?report=genbank&log$=prottop&blast_rank=39&RID=KD2R5136014) |
| alpha/beta fold hydrolase [Serratia quinivorans] | Serratia quinivorans | 489 | 489 | 100% | 2.00E-173 | 92.05 | 264 | [WP_261088244.1](https://www.ncbi.nlm.nih.gov/protein/WP_261088244.1?report=genbank&log$=prottop&blast_rank=40&RID=KD2R5136014) |
| alpha/beta fold hydrolase [Serratia grimesii] | Serratia grimesii | 489 | 489 | 100% | 2.00E-173 | 92.05 | 264 | [WP_186428409.1](https://www.ncbi.nlm.nih.gov/protein/WP_186428409.1?report=genbank&log$=prottop&blast_rank=41&RID=KD2R5136014) |
| alpha/beta fold hydrolase [Serratia plymuthica] | Serratia plymuthica | 489 | 489 | 100% | 2.00E-173 | 87.5 | 264 | [WP_434463010.1](https://www.ncbi.nlm.nih.gov/protein/WP_434463010.1?report=genbank&log$=prottop&blast_rank=42&RID=KD2R5136014) |
| alpha/beta hydrolase [Serratia plymuthica] | Serratia plymuthica | 489 | 489 | 100% | 3.00E-173 | 87.5 | 264 | [WP_063198482.1](https://www.ncbi.nlm.nih.gov/protein/WP_063198482.1?report=genbank&log$=prottop&blast_rank=43&RID=KD2R5136014) |
| alpha/beta fold hydrolase [Serratia grimesii] | Serratia grimesii | 489 | 489 | 100% | 3.00E-173 | 92.05 | 264 | [WP_037414937.1](https://www.ncbi.nlm.nih.gov/protein/WP_037414937.1?report=genbank&log$=prottop&blast_rank=44&RID=KD2R5136014) |
| alpha/beta fold hydrolase [Serratia grimesii] | Serratia grimesii | 489 | 489 | 100% | 3.00E-173 | 92.05 | 264 | [WP_413504138.1](https://www.ncbi.nlm.nih.gov/protein/WP_413504138.1?report=genbank&log$=prottop&blast_rank=45&RID=KD2R5136014) |
| alpha/beta fold hydrolase [Serratia grimesii] | Serratia grimesii | 489 | 489 | 100% | 3.00E-173 | 92.05 | 264 | [WP_261452987.1](https://www.ncbi.nlm.nih.gov/protein/WP_261452987.1?report=genbank&log$=prottop&blast_rank=46&RID=KD2R5136014) |
| MULTISPECIES: alpha/beta fold hydrolase [Serratia] | Serratia | 489 | 489 | 100% | 3.00E-173 | 87.5 | 264 | [WP_197912681.1](https://www.ncbi.nlm.nih.gov/protein/WP_197912681.1?report=genbank&log$=prottop&blast_rank=47&RID=KD2R5136014) |
| alpha/beta fold hydrolase [Serratia quinivorans] | Serratia quinivorans | 488 | 488 | 100% | 7.00E-173 | 91.29 | 264 | [WP_409326277.1](https://www.ncbi.nlm.nih.gov/protein/WP_409326277.1?report=genbank&log$=prottop&blast_rank=48&RID=KD2R5136014) |
| alpha/beta hydrolase [Serratia plymuthica] | Serratia plymuthica | 488 | 488 | 100% | 8.00E-173 | 87.12 | 264 | [WP_062870737.1](https://www.ncbi.nlm.nih.gov/protein/WP_062870737.1?report=genbank&log$=prottop&blast_rank=49&RID=KD2R5136014) |
| alpha/beta fold hydrolase [Serratia plymuthica] | Serratia plymuthica | 488 | 488 | 100% | 9.00E-173 | 87.12 | 264 | [WP_241920799.1](https://www.ncbi.nlm.nih.gov/protein/WP_241920799.1?report=genbank&log$=prottop&blast_rank=50&RID=KD2R5136014) |
| alpha/beta fold hydrolase [Serratia proteamaculans] | Serratia proteamaculans | 488 | 488 | 100% | 1.00E-172 | 91.67 | 264 | [WP_153860529.1](https://www.ncbi.nlm.nih.gov/protein/WP_153860529.1?report=genbank&log$=prottop&blast_rank=51&RID=KD2R5136014) |
| alpha/beta fold hydrolase [Serratia plymuthica] | Serratia plymuthica | 488 | 488 | 100% | 1.00E-172 | 87.5 | 264 | [WP_064799953.1](https://www.ncbi.nlm.nih.gov/protein/WP_064799953.1?report=genbank&log$=prottop&blast_rank=52&RID=KD2R5136014) |
| alpha/beta fold hydrolase [Serratia proteamaculans] | Serratia proteamaculans | 487 | 487 | 100% | 1.00E-172 | 91.67 | 264 | [WP_174352401.1](https://www.ncbi.nlm.nih.gov/protein/WP_174352401.1?report=genbank&log$=prottop&blast_rank=53&RID=KD2R5136014) |
| alpha/beta fold hydrolase [Serratia quinivorans] | Serratia quinivorans | 487 | 487 | 100% | 2.00E-172 | 91.67 | 264 | [WP_261106606.1](https://www.ncbi.nlm.nih.gov/protein/WP_261106606.1?report=genbank&log$=prottop&blast_rank=54&RID=KD2R5136014) |
| alpha/beta fold hydrolase [Serratia quinivorans] | Serratia quinivorans | 487 | 487 | 100% | 2.00E-172 | 91.29 | 264 | [WP_112362920.1](https://www.ncbi.nlm.nih.gov/protein/WP_112362920.1?report=genbank&log$=prottop&blast_rank=55&RID=KD2R5136014) |
| alpha/beta fold hydrolase [Serratia sp. BIGb0163] | Serratia sp. BIGb0163 | 486 | 486 | 100% | 3.00E-172 | 91.29 | 264 | [WP_259189191.1](https://www.ncbi.nlm.nih.gov/protein/WP_259189191.1?report=genbank&log$=prottop&blast_rank=56&RID=KD2R5136014) |
| alpha/beta fold hydrolase [Serratia quinivorans] | Serratia quinivorans | 486 | 486 | 100% | 3.00E-172 | 91.29 | 264 | [WP_261413833.1](https://www.ncbi.nlm.nih.gov/protein/WP_261413833.1?report=genbank&log$=prottop&blast_rank=57&RID=KD2R5136014) |
| alpha/beta fold hydrolase [Serratia quinivorans] | Serratia quinivorans | 486 | 486 | 100% | 3.00E-172 | 90.91 | 264 | [WP_368460744.1](https://www.ncbi.nlm.nih.gov/protein/WP_368460744.1?report=genbank&log$=prottop&blast_rank=58&RID=KD2R5136014) |
| alpha/beta fold hydrolase [Serratia grimesii] | Serratia grimesii | 486 | 486 | 100% | 3.00E-172 | 91.67 | 264 | [WP_278431507.1](https://www.ncbi.nlm.nih.gov/protein/WP_278431507.1?report=genbank&log$=prottop&blast_rank=59&RID=KD2R5136014) |
| MULTISPECIES: alpha/beta fold hydrolase [Serratia] | Serratia | 486 | 486 | 100% | 3.00E-172 | 91.29 | 264 | [WP_115184732.1](https://www.ncbi.nlm.nih.gov/protein/WP_115184732.1?report=genbank&log$=prottop&blast_rank=60&RID=KD2R5136014) |
| alpha/beta hydrolase [Serratia plymuthica] | Serratia plymuthica | 486 | 486 | 100% | 4.00E-172 | 87.12 | 264 | [WP_197927314.1](https://www.ncbi.nlm.nih.gov/protein/WP_197927314.1?report=genbank&log$=prottop&blast_rank=61&RID=KD2R5136014) |
| MULTISPECIES: alpha/beta fold hydrolase [unclassified Serratia (in: enterobacteria)] | unclassified Serratia (in: enterobacteria) | 486 | 486 | 100% | 4.00E-172 | 90.91 | 264 | [WP_209633221.1](https://www.ncbi.nlm.nih.gov/protein/WP_209633221.1?report=genbank&log$=prottop&blast_rank=62&RID=KD2R5136014) |
| alpha/beta hydrolase [Serratia sp. PAMC26656] | Serratia sp. PAMC26656 | 486 | 486 | 100% | 6.00E-172 | 87.12 | 264 | [WP_199639304.1](https://www.ncbi.nlm.nih.gov/protein/WP_199639304.1?report=genbank&log$=prottop&blast_rank=63&RID=KD2R5136014) |
| alpha/beta fold hydrolase [Serratia quinivorans] | Serratia quinivorans | 486 | 486 | 100% | 7.00E-172 | 91.29 | 264 | [WP_261164947.1](https://www.ncbi.nlm.nih.gov/protein/WP_261164947.1?report=genbank&log$=prottop&blast_rank=64&RID=KD2R5136014) |
| MULTISPECIES: alpha/beta fold hydrolase [Serratia] | Serratia | 486 | 486 | 100% | 7.00E-172 | 91.29 | 264 | [WP_017892747.1](https://www.ncbi.nlm.nih.gov/protein/WP_017892747.1?report=genbank&log$=prottop&blast_rank=65&RID=KD2R5136014) |
| alpha/beta fold hydrolase [Serratia quinivorans] | Serratia quinivorans | 485 | 485 | 100% | 1.00E-171 | 91.29 | 264 | [WP_261420202.1](https://www.ncbi.nlm.nih.gov/protein/WP_261420202.1?report=genbank&log$=prottop&blast_rank=66&RID=KD2R5136014) |
| MULTISPECIES: alpha/beta fold hydrolase [Serratia] | Serratia | 485 | 485 | 100% | 1.00E-171 | 87.12 | 264 | [WP_013812793.1](https://www.ncbi.nlm.nih.gov/protein/WP_013812793.1?report=genbank&log$=prottop&blast_rank=67&RID=KD2R5136014) |
| alpha/beta fold hydrolase [Serratia quinivorans] | Serratia quinivorans | 484 | 484 | 100% | 1.00E-171 | 90.91 | 264 | [WP_454889461.1](https://www.ncbi.nlm.nih.gov/protein/WP_454889461.1?report=genbank&log$=prottop&blast_rank=68&RID=KD2R5136014) |
| alpha/beta fold hydrolase [Serratia proteamaculans] | Serratia proteamaculans | 484 | 484 | 100% | 2.00E-171 | 90.91 | 264 | [WP_413476930.1](https://www.ncbi.nlm.nih.gov/protein/WP_413476930.1?report=genbank&log$=prottop&blast_rank=69&RID=KD2R5136014) |
| alpha/beta fold hydrolase [Serratia proteamaculans] | Serratia proteamaculans | 484 | 484 | 100% | 2.00E-171 | 90.91 | 264 | [WP_413506982.1](https://www.ncbi.nlm.nih.gov/protein/WP_413506982.1?report=genbank&log$=prottop&blast_rank=70&RID=KD2R5136014) |
| alpha/beta fold hydrolase [Serratia quinivorans] | Serratia quinivorans | 484 | 484 | 100% | 2.00E-171 | 90.91 | 264 | [WP_218216917.1](https://www.ncbi.nlm.nih.gov/protein/WP_218216917.1?report=genbank&log$=prottop&blast_rank=71&RID=KD2R5136014) |
| MULTISPECIES: alpha/beta fold hydrolase [Serratia] | Serratia | 484 | 484 | 100% | 2.00E-171 | 90.91 | 264 | [WP_261283277.1](https://www.ncbi.nlm.nih.gov/protein/WP_261283277.1?report=genbank&log$=prottop&blast_rank=72&RID=KD2R5136014) |
| MULTISPECIES: alpha/beta hydrolase [unclassified Serratia (in: enterobacteria)] | unclassified Serratia (in: enterobacteria) | 484 | 484 | 100% | 3.00E-171 | 86.36 | 264 | [WP_330761983.1](https://www.ncbi.nlm.nih.gov/protein/WP_330761983.1?report=genbank&log$=prottop&blast_rank=73&RID=KD2R5136014) |
| alpha/beta fold hydrolase [Serratia quinivorans] | Serratia quinivorans | 484 | 484 | 100% | 3.00E-171 | 91.29 | 264 | [WP_261122412.1](https://www.ncbi.nlm.nih.gov/protein/WP_261122412.1?report=genbank&log$=prottop&blast_rank=74&RID=KD2R5136014) |
| alpha/beta fold hydrolase [Serratia quinivorans] | Serratia quinivorans | 484 | 484 | 100% | 3.00E-171 | 90.91 | 264 | [WP_261129005.1](https://www.ncbi.nlm.nih.gov/protein/WP_261129005.1?report=genbank&log$=prottop&blast_rank=75&RID=KD2R5136014) |
| alpha/beta fold hydrolase [Serratia quinivorans] | Serratia quinivorans | 484 | 484 | 100% | 3.00E-171 | 90.53 | 264 | [WP_261407999.1](https://www.ncbi.nlm.nih.gov/protein/WP_261407999.1?report=genbank&log$=prottop&blast_rank=76&RID=KD2R5136014) |
| alpha/beta fold hydrolase [Serratia quinivorans] | Serratia quinivorans | 484 | 484 | 100% | 3.00E-171 | 91.29 | 264 | [WP_261409893.1](https://www.ncbi.nlm.nih.gov/protein/WP_261409893.1?report=genbank&log$=prottop&blast_rank=77&RID=KD2R5136014) |
| alpha/beta fold hydrolase [Serratia proteamaculans] | Serratia proteamaculans | 484 | 484 | 100% | 4.00E-171 | 90.91 | 264 | [WP_219016106.1](https://www.ncbi.nlm.nih.gov/protein/WP_219016106.1?report=genbank&log$=prottop&blast_rank=78&RID=KD2R5136014) |
| MULTISPECIES: alpha/beta fold hydrolase [Serratia] | Serratia | 484 | 484 | 100% | 4.00E-171 | 90.91 | 264 | [WP_012145038.1](https://www.ncbi.nlm.nih.gov/protein/WP_012145038.1?report=genbank&log$=prottop&blast_rank=79&RID=KD2R5136014) |
| alpha/beta fold hydrolase [Serratia inhibens] | Serratia inhibens | 484 | 484 | 100% | 4.00E-171 | 86.74 | 264 | [WP_346828195.1](https://www.ncbi.nlm.nih.gov/protein/WP_346828195.1?report=genbank&log$=prottop&blast_rank=80&RID=KD2R5136014) |
| alpha/beta fold hydrolase [Serratia proteamaculans] | Serratia proteamaculans | 483 | 483 | 100% | 5.00E-171 | 90.53 | 264 | [WP_261284508.1](https://www.ncbi.nlm.nih.gov/protein/WP_261284508.1?report=genbank&log$=prottop&blast_rank=81&RID=KD2R5136014) |
| alpha/beta fold hydrolase [Serratia proteamaculans] | Serratia proteamaculans | 483 | 483 | 100% | 9.00E-171 | 90.15 | 264 | [WP_261254916.1](https://www.ncbi.nlm.nih.gov/protein/WP_261254916.1?report=genbank&log$=prottop&blast_rank=82&RID=KD2R5136014) |
| alpha/beta fold hydrolase [Serratia proteamaculans] | Serratia proteamaculans | 483 | 483 | 100% | 9.00E-171 | 90.53 | 264 | [WP_207976992.1](https://www.ncbi.nlm.nih.gov/protein/WP_207976992.1?report=genbank&log$=prottop&blast_rank=83&RID=KD2R5136014) |
| alpha/beta fold hydrolase [Serratia proteamaculans] | Serratia proteamaculans | 483 | 483 | 100% | 9.00E-171 | 90.15 | 264 | [WP_261423793.1](https://www.ncbi.nlm.nih.gov/protein/WP_261423793.1?report=genbank&log$=prottop&blast_rank=84&RID=KD2R5136014) |
| alpha/beta fold hydrolase [Serratia quinivorans] | Serratia quinivorans | 483 | 483 | 100% | 1.00E-170 | 90.91 | 264 | [WP_261151956.1](https://www.ncbi.nlm.nih.gov/protein/WP_261151956.1?report=genbank&log$=prottop&blast_rank=85&RID=KD2R5136014) |
| alpha/beta fold hydrolase [Serratia proteamaculans] | Serratia proteamaculans | 483 | 483 | 100% | 1.00E-170 | 90.53 | 264 | [WP_129934742.1](https://www.ncbi.nlm.nih.gov/protein/WP_129934742.1?report=genbank&log$=prottop&blast_rank=86&RID=KD2R5136014) |
| alpha/beta fold hydrolase [Serratia proteamaculans] | Serratia proteamaculans | 482 | 482 | 100% | 2.00E-170 | 90.15 | 264 | [WP_262207927.1](https://www.ncbi.nlm.nih.gov/protein/WP_262207927.1?report=genbank&log$=prottop&blast_rank=87&RID=KD2R5136014) |
| alpha/beta fold hydrolase [Serratia proteamaculans] | Serratia proteamaculans | 482 | 482 | 100% | 2.00E-170 | 90.15 | 264 | [WP_413480696.1](https://www.ncbi.nlm.nih.gov/protein/WP_413480696.1?report=genbank&log$=prottop&blast_rank=88&RID=KD2R5136014) |
| alpha/beta fold hydrolase [Serratia proteamaculans] | Serratia proteamaculans | 481 | 481 | 100% | 3.00E-170 | 90.15 | 264 | [WP_413514315.1](https://www.ncbi.nlm.nih.gov/protein/WP_413514315.1?report=genbank&log$=prottop&blast_rank=89&RID=KD2R5136014) |
| alpha/beta fold hydrolase [Serratia sp. BW106] | Serratia sp. BW106 | 481 | 481 | 100% | 3.00E-170 | 90.15 | 264 | [WP_099061908.1](https://www.ncbi.nlm.nih.gov/protein/WP_099061908.1?report=genbank&log$=prottop&blast_rank=90&RID=KD2R5136014) |
| alpha/beta fold hydrolase [Serratia proteamaculans] | Serratia proteamaculans | 481 | 481 | 100% | 5.00E-170 | 90.15 | 264 | [MGO2154338.1](https://www.ncbi.nlm.nih.gov/protein/MGO2154338.1?report=genbank&log$=prottop&blast_rank=91&RID=KD2R5136014) |
| alpha/beta fold hydrolase [Serratia proteamaculans] | Serratia proteamaculans | 481 | 481 | 100% | 6.00E-170 | 90.15 | 264 | [WP_261462536.1](https://www.ncbi.nlm.nih.gov/protein/WP_261462536.1?report=genbank&log$=prottop&blast_rank=92&RID=KD2R5136014) |
| alpha/beta fold hydrolase [Serratia proteamaculans] | Serratia proteamaculans | 480 | 480 | 100% | 8.00E-170 | 90.15 | 264 | [WP_261417139.1](https://www.ncbi.nlm.nih.gov/protein/WP_261417139.1?report=genbank&log$=prottop&blast_rank=93&RID=KD2R5136014) |
| alpha/beta hydrolase [Serratia liquefaciens] | Serratia liquefaciens | 475 | 475 | 88% | 3.00E-168 | 98.71 | 232 | [MDU3890911.1](https://www.ncbi.nlm.nih.gov/protein/MDU3890911.1?report=genbank&log$=prottop&blast_rank=94&RID=KD2R5136014) |
| alpha/beta hydrolase [Serratia marcescens] | Serratia marcescens | 474 | 474 | 100% | 2.00E-167 | 84.85 | 264 | [HGM5336213.1](https://www.ncbi.nlm.nih.gov/protein/HGM5336213.1?report=genbank&log$=prottop&blast_rank=95&RID=KD2R5136014) |
| alpha/beta fold hydrolase [Serratia marcescens] | Serratia marcescens | 474 | 474 | 100% | 2.00E-167 | 84.47 | 264 | [WP_421084949.1](https://www.ncbi.nlm.nih.gov/protein/WP_421084949.1?report=genbank&log$=prottop&blast_rank=96&RID=KD2R5136014) |
| alpha/beta fold hydrolase [Serratia nevei] | Serratia nevei | 474 | 474 | 100% | 2.00E-167 | 84.85 | 264 | [WP_442994458.1](https://www.ncbi.nlm.nih.gov/protein/WP_442994458.1?report=genbank&log$=prottop&blast_rank=97&RID=KD2R5136014) |
| alpha/beta hydrolase [Serratia nevei] | Serratia nevei | 474 | 474 | 100% | 3.00E-167 | 84.85 | 264 | [WP_342121584.1](https://www.ncbi.nlm.nih.gov/protein/WP_342121584.1?report=genbank&log$=prottop&blast_rank=98&RID=KD2R5136014) |
| MULTISPECIES: alpha/beta hydrolase [Serratia] | Serratia | 474 | 474 | 100% | 3.00E-167 | 84.85 | 264 | [WP_060440011.1](https://www.ncbi.nlm.nih.gov/protein/WP_060440011.1?report=genbank&log$=prottop&blast_rank=99&RID=KD2R5136014) |
| alpha/beta hydrolase [Serratia marcescens] | Serratia marcescens | 473 | 473 | 100% | 4.00E-167 | 84.85 | 264 | [GJK49481.1](https://www.ncbi.nlm.nih.gov/protein/GJK49481.1?report=genbank&log$=prottop&blast_rank=100&RID=KD2R5136014) |

Table S2. Effect of EDTA and metal ions on SlDefH activity. Esterase activity was measured using p-NPA and dehalogenase activity was measured using fluoroacetate as substrate (± standard deviation).

| **Metal ion or EDTA (5 mM)** | **Esterase activity (%)** | **Dehalogenase activity (%)** |
| --- | --- | --- |
| **No Metal Control** | 100.0 ± 1.5 | 100.0 ± 2.2 |
| **Mg^2+^** | 92.3 ± 2.9 | 93.7 ± 0.5 |
| **Ca^2+^** | 119.3 ± 2.3 | 99.1 ± 4.1 |
| **Co^2+^** | 92.5 ± 0.8 | 92.4 ± 2.3 |
| **Fe^3+^** | 88.0 ± 1.4 | 82.3 ± 5.3 |
| **EDTA** | 101.3 ± 2.3 | 99.8 ± 3.2 |

Figure S1. Glycolate standard curve (errors bars show standard deviation)


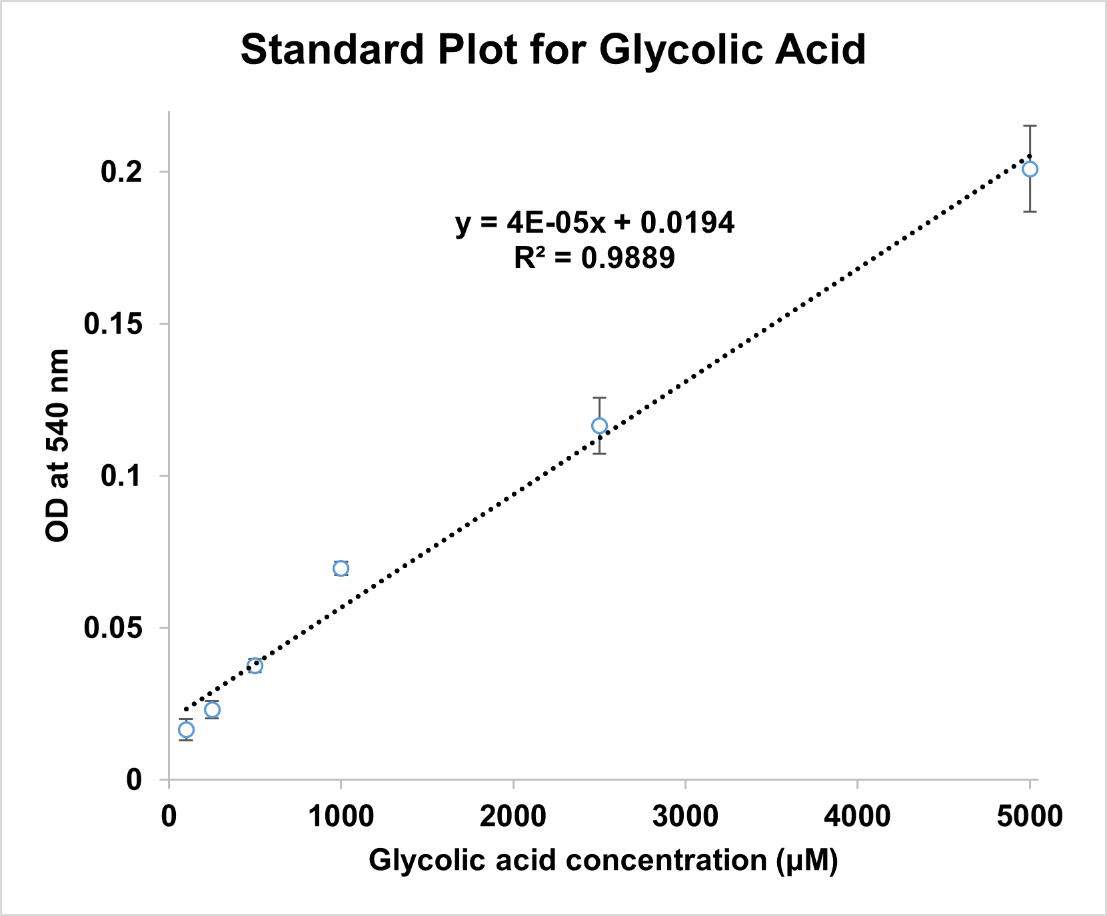


Figure S2. Calibration curve for fluoride ion (error bars show standard deviation).


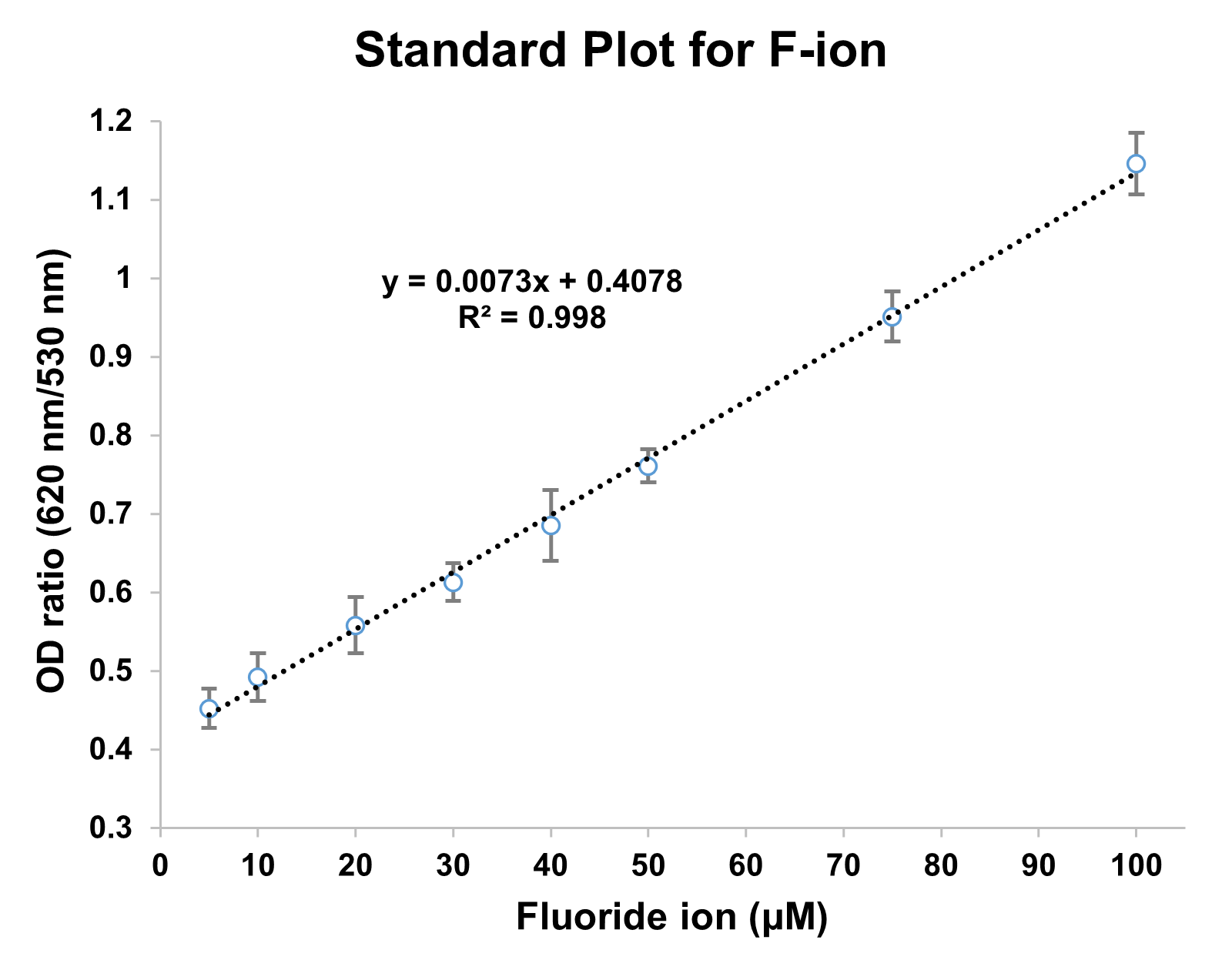


Figure S3. GC-MS of authentic silylated homoserine


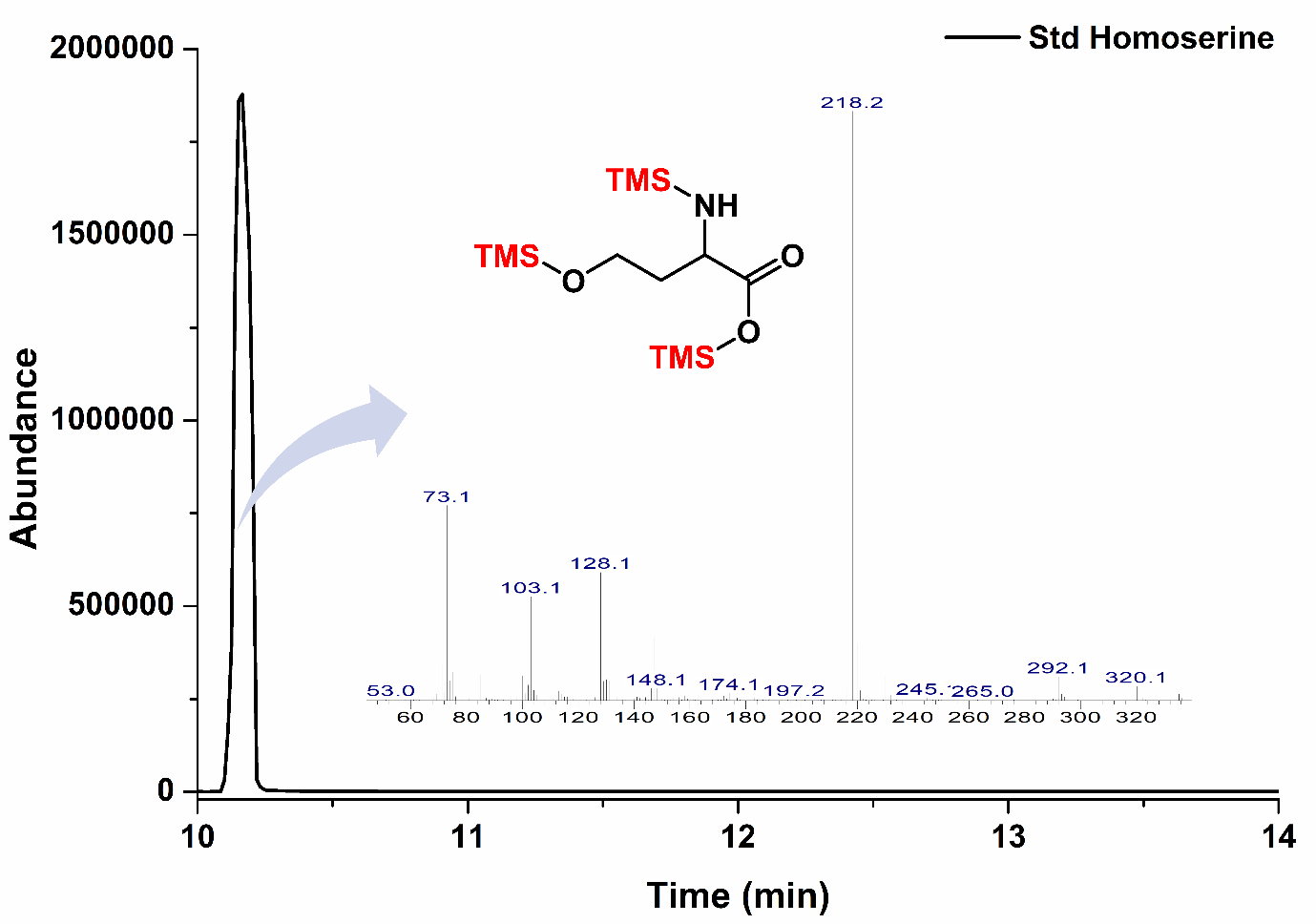


Figure S4. Dehalogenation of haloacetates by SLDH.





Figure S5. Mass spectrum of silylated N-formylmaleamic acid.


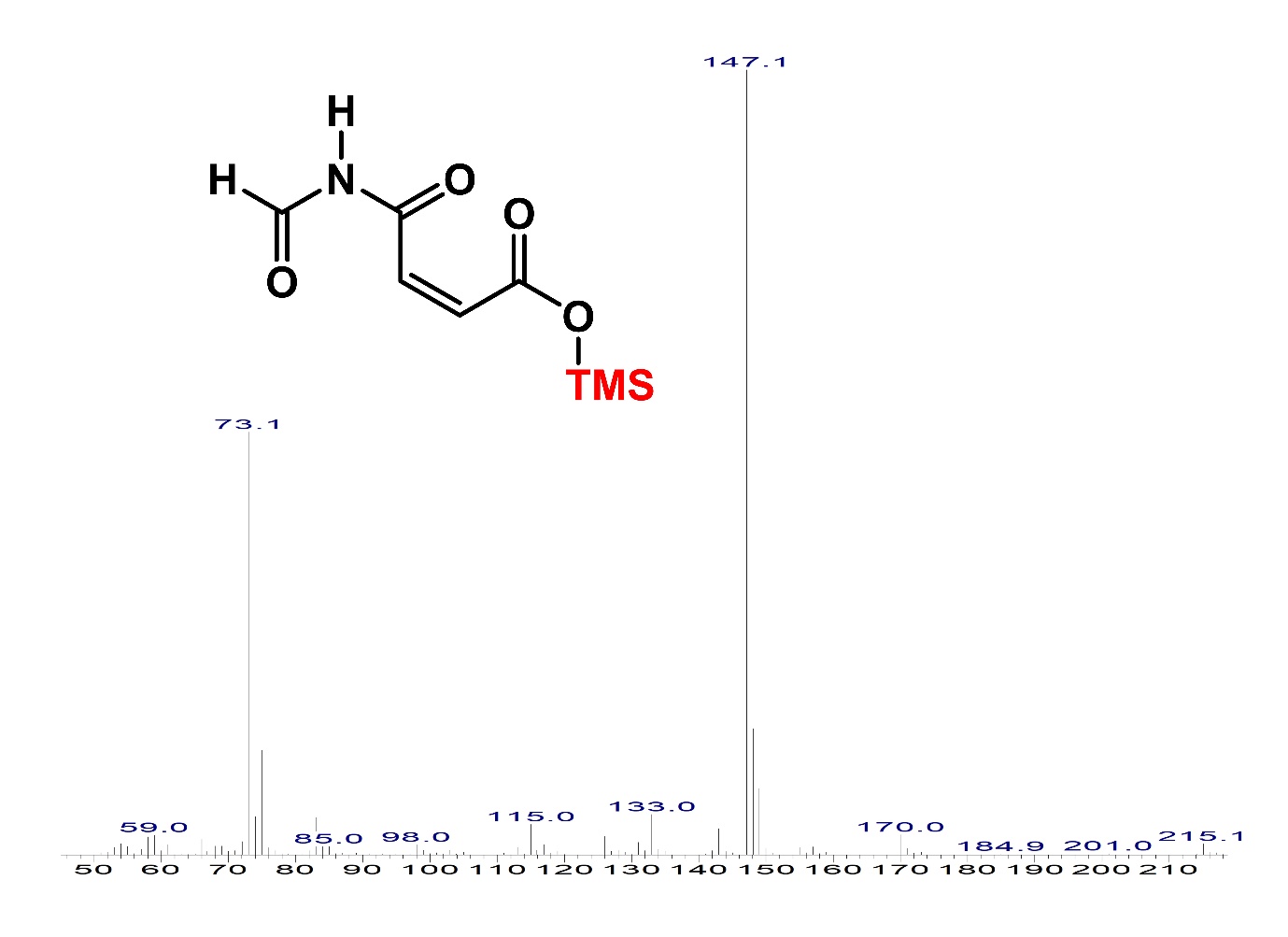


Figure S6. Temperature and pH optima for esterase and dehalogenase activities.


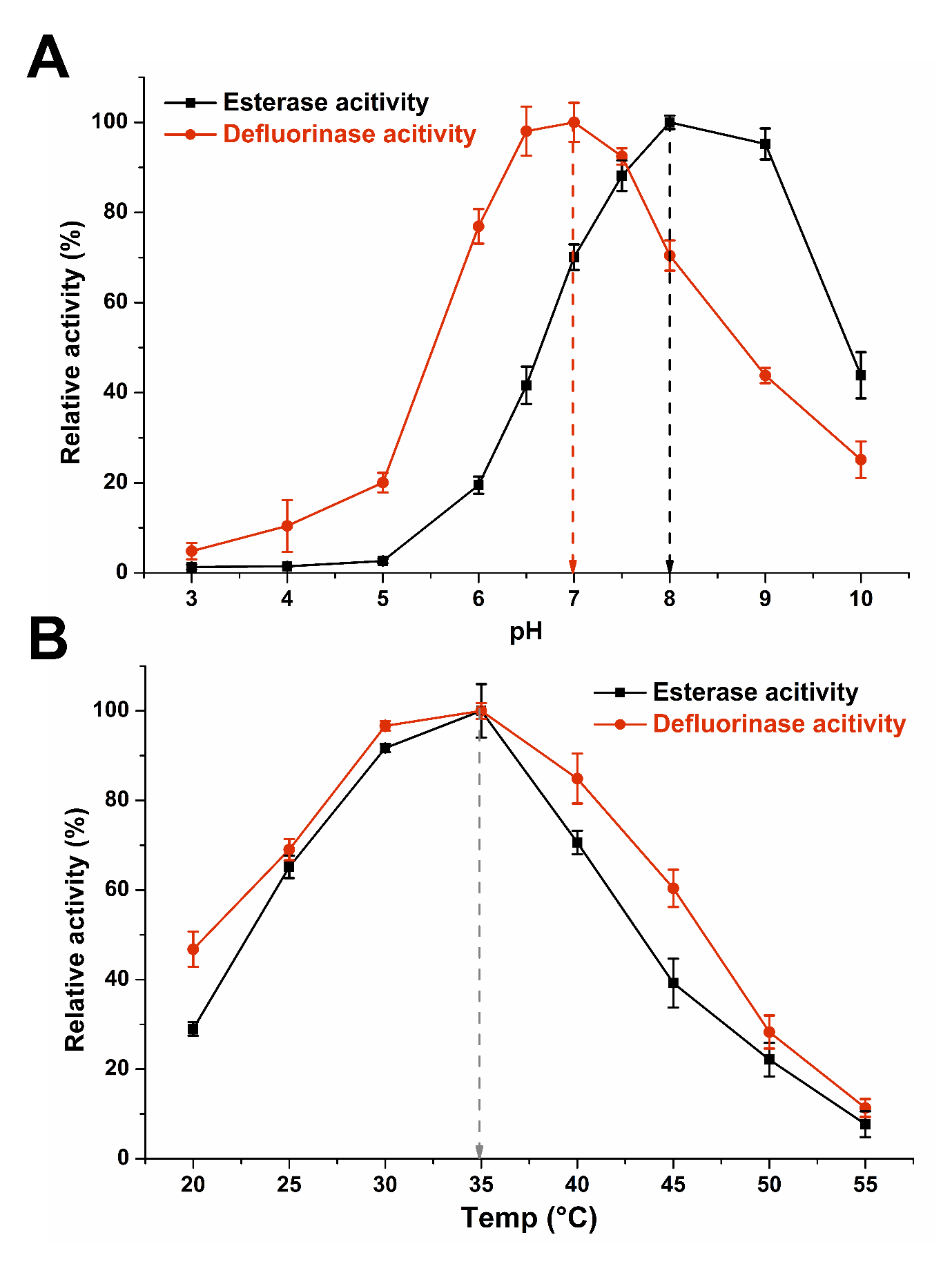


Figure S7. Kinetic analysis of SlDefH wild type and SlDefH-S101D. Assays were conducted measuring dehalogenase (A) and esterase (B) activities. Michaelis–Menten fits for dehalogenase and esterase activities of SlDefH and SlDefH-S101D. Symbols represent experimental data points (mean ± SD), and solid lines indicate nonlinear regression fits. Corresponding kinetic parameters and 95% confidence intervals (CI) are provided in the tables below.


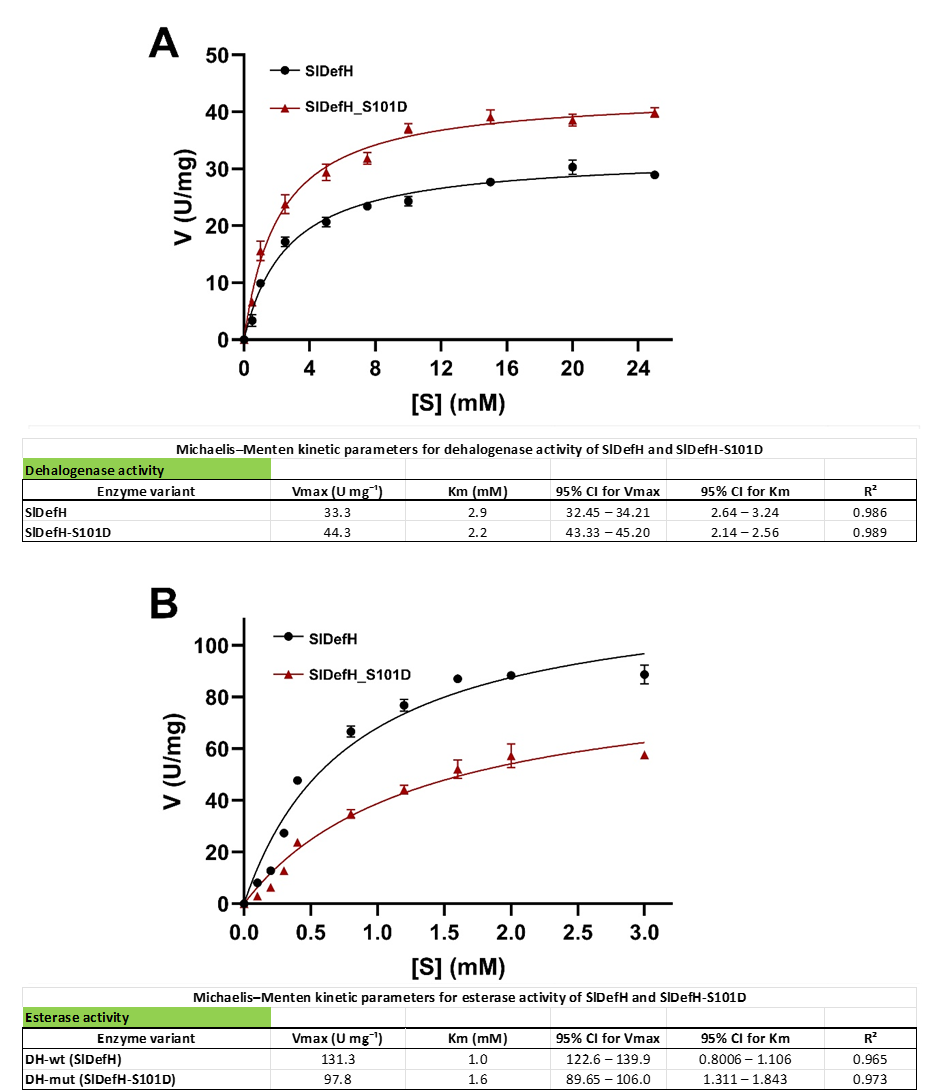


Figure S8. Sequence alignments of SLDH homologues showing the presumed catalytically important amino acid residues.


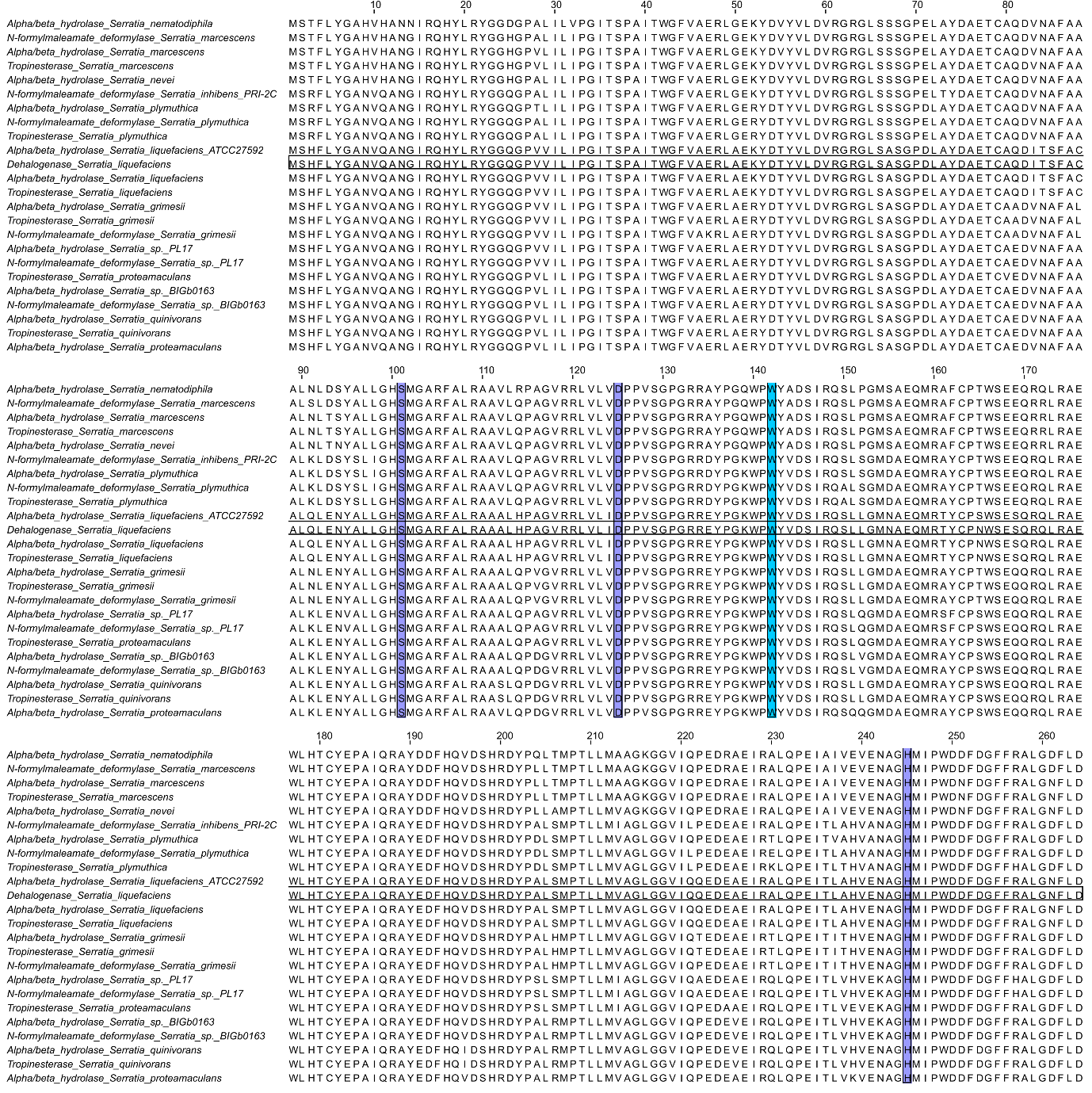

Supplement: Supplementary file 1 — DOCX (2.25 MB) [file 253_2026_13829_MOESM1_ESM.docx]
